# MicroRNA Expression Changes in Women with Breast Cancer Stratified by DNA Repair Capacity Levels

**DOI:** 10.1155/2019/7820275

**Published:** 2019-05-05

**Authors:** Jarline Encarnación-Medina, Carmen Ortiz, Ralphdy Vergne, Luis Padilla, Jaime Matta

**Affiliations:** ^1^Basic Sciences Department, Division of Pharmacology & Toxicology and Cancer Biology, Ponce Research Institute, Ponce 00716-2348, PR, USA; ^2^Biology Department, University of Puerto Rico at Ponce, Ponce 00716-9996, PR, USA

## Abstract

Breast cancer (BC) is the most commonly diagnosed cancer in women worldwide and is the leading cause of death among Hispanic women. Previous studies have shown that women with a low DNA repair capacity (DRC), measured through the nucleotide excision repair (NER) pathway, have an increased BC risk. Moreover, we previously reported an association between DRC levels and the expression of the microRNA (miRNA) let-7b in BC patients. MiRNAs can induce genomic instability by affecting the cell's DNA damage response while influencing the cancer pathobiology. The aim of this pilot study is to identify plasma miRNAs related to variations in DRC levels in BC cases.* Hypothesis*. Our hypothesis consists in testing whether DRC levels can be correlated with miRNA expression levels.* Methods*. Plasma samples were selected from 56 (27 cases and 29 controls) women recruited as part of our BC cohort. DRC values were measured in lymphocytes using the host-cell reactivation assay. The samples were divided into two categories: low (≤3.8%) and high (>3.8%) DRC levels. MiRNAs were extracted to perform an expression profile analysis.* Results*. Forty miRNAs were identified to be BC-related (p<0.05, MW), while 18 miRNAs were found to be differentially expressed among BC cases and controls with high and low DRC levels (p<0.05, KW). Among these candidates are miR-299-5p, miR-29b-3p, miR-302c-3p, miR-373-3p, miR-636, miR-331-5p, and miR-597-5p. Correlation analyses revealed that 4 miRNAs were negatively correlated within BC cases with low DRC (p<0.05, Spearman's correlation). Results from multivariate analyses revealed that the clinicopathological characteristics may not have a direct effect on specific miRNA expression.* Conclusion*. This pilot study provides evidence of four miRNAs that are negatively regulated in BC cases with low DRC levels. Additional studies are needed in order to have a complete framework regarding the overall DRC levels, miRNA expression profiles, and tumor characteristics.

## 1. Introduction

The American Cancer Society estimates that in 2019, 268,600 new breast cancer (BC) cases and 41,760 cancer-related deaths will occur in the US [1]. The Center of Disease Control (CDC) reported that BC is the second cause of death among women in the US and the leading cause of death among Hispanic women. In the US and Puerto Rico (PR), BC now accounts for approximately 30% of all new cancers in women [2, 3]. In PR, 2,205 new BC cases and 444 BC deaths were reported by the Puerto Rico Cancer Registry in 2015 [3].

Cancer is a complex disease with genetic, epigenetic, and environmental risk factors. Genomic instability is a known hallmark of cancer as described by Hanahan and Weinberg (2011) [4]. Dysregulation of various DNA repair pathways contributes to this genomic instability due to inability of the cell to repair certain types of DNA damage [5, 6]. Defective DNA repair measured in blood cells has been identified as a risk factor for different types of cancer [7–9], including BC [9]. At least 5 DNA repair pathways have been described which contain approximately 200 DNA repair genes [10–12]. Nucleotide excision repair (NER) is a very versatile repair pathway that involves around 30 proteins that act to replace damaged nucleotides [13]. NER is the predominant mechanism by which bulky DNA adducts are repaired. These can be formed by multiple sources of DNA damage including UV light, exogenous chemicals, and chemotherapeutic agents like cisplatin [13]. Previous studies from our laboratory demonstrate the critical importance of the overall DRC levels for BC risk through the NER pathway using lymphocytes as surrogate markers [14, 15]. Recently, our laboratory showed that there is substantial variability in overall DRC levels among the four principal molecular BC subtypes and that women with triple-negative (TN) BC have the lowest DRC [16]. These findings confirm the importance of the NER pathway in sporadic BC and highlight the need for more research to understand how changes in DRC levels are associated with multiple endpoints in the complex process of BC carcinogenesis.

The epigenome has been proposed as an intermediary between genotype and phenotype [17]. Hence, the use of epigenetic analysis holds substantial promise for identifying mechanisms through which genetic and environmental factors contribute to disease risk [18]. Epigenetic changes have been associated with DRC levels once the BC malignancy is developed. For example, plasma levels of let-7b microRNA (miRNA) have been associated with high DRC levels in women with BC [19]. MiRNAs are endogenous, short (19–24 nucleotides) non-protein-coding RNAs that regulate gene expression at the posttranscriptional level via binding to 3′-untranslated regions of protein-coding transcripts [20]. Their aberrant expression in peripheral blood has been associated with different types of cancer [21]. MiRNAs are pleiotropic in terms of functions and have been shown to regulate the expression of a broad range of genes involved in cancer. However, very little is known of their role in the regulation of DRC levels in BC. Therefore, the main objectives of this pilot study were to (1) identify miRNAs that are related to BC, (2) identify miRNAs that are correlated with DRC levels in women with BC, and (3) test whether the clinicopathological characteristics from the women studied contribute to DRC levels.

## 2. Materials and Methods

*Use of Human Subjects*. The Ponce Health Sciences University Institutional Review Board approved this study (IRB #130207-JM). Each participant signed an Informed Consent form, providing permission to collect a blood sample and to review their pathology reports. All participants completed a 7-page epidemiological questionnaire requesting demographic data and BC risk factors which was administered by the study nurse.

*Patient Recruitment*. Participants in this study were selected from our BC cohort recruited from 2006 to 2013 (1,187 women with and without BC) as described in Matta et al. 2012 [22]. For each participant, blood samples were collected along with epidemiological data through a questionnaire. Plasma and lymphocytes were isolated from the blood samples. BC cases included in this study were recently diagnosed, treatment-naïve (had not received blood transfusions, chemotherapy, or radiotherapy) patients with primary breast tumors. Controls (women without BC) were required to have a normal breast examination performed by a primary care physician and a normal mammography, 6 months prior to study enrolment, and no prior history of any cancer type. Pathology reports from BC cases were obtained for diagnosis confirmation and collection of clinicopathological variables such as tumor size and grade, type of cancer, hormone receptor status, and other clinically relevant information. Using the hormone receptor status data for estrogen (ER) and progesterone (PR) receptors, along with HER2, the tumors of the BC cases were classified into four principal molecular subtypes: luminal A (ER+, PR+, HER2−), luminal B (ER+, PR+, HER2+), HER2+ (ER−, PR−, HER2+), and triple negative (TN) (ER−, PR−, HER2−). For this pilot study, 56 participants, 27 BC cases and 29 controls, were selected from our BC cohort including cases and controls with high and low DRC.

*DNA Repair Capacity Measurements*. Peripheral blood lymphocytes were separated, purified, and grown from each patient sample, as previously reported [9, 22]. The isolated lymphocytes were used as surrogate markers of the patients' overall DRC [23, 24], measured using the host-cell reactivation (HCR) assay. This assay allows for the measurement of in vivo DRC, as previously published [9, 25–28]. The lymphocytes' capacity to repair the foreign DNA was measured via HCR [25] within a specific time frame (40 h) that mirrored the true cellular process [24]. Results reflected the cells' inherent DRC, measured primarily in terms of their NER pathway activity [25]. Briefly, the lymphocytes were transfected with a plasmid, previously damaged with UVC light, containing the luciferase reporter gene. To calculate DRC, the luciferase activity after repair of the UVC-damaged plasmid DNA was compared with the undamaged plasmid DNA. The amount of residual luciferase remaining after the allotted repair time (activity in luminescence units) was a percentage that represented the amount of the individuals' DRC. DRC levels were established as previously described using the cut-off of ≥3.8% for high DRC and <3.8% for low DRC [19].

*MicroRNA Expression Profile*. MicroRNA expression profiling was performed utilizing the TaqMan Array Human MicroRNA A Cards v 2.0 (Applied Biosystems). miRNAs were extracted from the 56 plasma samples using the Ambion* mir*Vana miRNA Isolation Kit (Life Technologies; Grand Island, NY). RNA concentration and quality were determined using a NanoDrop 1000; 0.5–1 mg total RNA was reverse-transcribed with pools of miRNA-specific RT primers. A preamplification step using Megaplex PreAmp Primers, Human Pool A v2.1, was performed to increase sensitivity. Single-stranded cDNA was synthesized from 200 ng of total RNA in 8 Multiplex RT primer pool reactions containing stem-looped RT primers that were specific to mature miRNA. The resulting cDNA samples were diluted, combined with TaqMan Universal PCR Master Mix (Applied Biosystems), and then loaded onto the TaqMan Array. Quantitative PCR was carried out under the following thermocycler conditions: 30 min at 16°C, 30 min at 42°C, 5 min at 85°C and then held at 4°C. Experimental Ct fluorescence evaluation was performed by testing for experimental outliers, and only cycles between 20 and 40 Ct were considered. Sample normalization was performed using U6 as endogenous miRNA. All the miRNA expression experiments were performed at the Molecular Genomics Core, H. Lee Moffitt Cancer Center (Tampa, Florida).

*Statistical Analyses*. To assess mean expression differences between BC cases and controls, a Mann–Whitney test was performed. Comparisons among miRNA expression levels among groups were performed using a Kruskal–Wallis test. The hierarchical microRNA clustering was performed using Morpheus heatmap generator [29] and tested for correlations using the Pearson's correlation test. Proportion analyses were performed using cross-tables, and differences were detected using a chi-square test. Significant correlations between miRNA expression and DRC levels were evaluated using the Spearman's correlation. Statistical significance was defined using a p-value of 0.05 or less based on a two-tailed test. Analyses were performed using Prism 8 (GraphPad Software; San Diego, CA) and Minitab® 18 (Minitab Inc.; State College, PA).

*Multivariate Analysis*. The principal component (PC) algorithm creates a series of artificial coordinates using the original matrix data, to localize the samples relative to each other. This analysis is best interpreted using a score plot graph. This graph consolidates the sample cluster formation where the closer the samples are located, the less the variability among them. The PC matrix for this study was constructed using the miRNA relative expression values, excluding the miRNAs with missing values from the analysis. The PC matrix was constructed with these miRNAs along with some of the epidemiological and clinicopathological information for each participant including case or control classification, DRC levels (high and low), tumor grade, and molecular subtype classification. Multivariate analyses and PC graphs were created in Minitab® 18 (Minitab Inc.; State College, PA).

## 3. Results

### 3.1. Epidemiological and Clinicopathological Variables

As an initial analysis, differences between cases and controls regarding known BC risk factors were assessed for body mass index (BMI), pregnancy, parity, breastfeeding practices, use of oral contraceptives, regularity of menstrual periods, history of endometriosis, hysterectomy, age of hysterectomy, oophorectomy, age of oophorectomy, family history of cancer, and BC history. Cross-table analyses showed no significant differences in the study cohort stratified by these variables (p>0.05, Pearson's chi-square test) (Table 1). However, statistically significant differences were found between BC cases and controls stratified by age (p=0.0239), age of menarche (p=0.0001), and use of hormone replacement therapy (p=0.0420). Differences in clinicopathological characteristics were evaluated among BC cases stratified in terms of low (<3.8%) and high (≥3.8%) DRC levels (Table 2). The clinicopathological characteristics analyzed were hormone receptor status (estrogen: ER, progesterone: PR, and HER2), Ki-67 expression, tumor grade and site, and type of BC. No significant differences were found regarding any of these clinicopathological characteristics between BC cases with low and high DRC levels (Table 2). Using the hormone receptor status information, BC cases were stratified into the four principal molecular subtypes: luminal A (ER+, PR+, HER2−), luminal B (ER+, PR+, HER2+), HER2+ (ER−, PR−, HER2+), and triple negative (ER−, PR−, HER2−). However, no significant differences were observed among groups (p>0.05, Pearson's chi-square test) (Table 2).

### 3.2. Identification of Breast Cancer-Related MicroRNAs

A case-control stratification was performed to identify the miRNAs that were significantly related to BC in Hispanic women. The qualitative aspect of the data is illustrated using hierarchical clustering where only the rows were taken into consideration to assure that the generated heatmap captured the miRNA expression from BC cases and controls separately (Figure S1). The heatmap proportion with higher miRNA expression abundance was composed of BC cases (Figure S1(a)) when compared with the control group (Figure S1(b)). A correlation analysis was performed to measure the distance between the expression profiles from BC cases and controls and to assess any linear relationship between expression patterns; however, no significant correlation was found (data not shown) (p>0.05, Pearson's chi-square test).

In general, the miRNA expression values obtained from this experiment cannot be defined in terms of a normal or Gaussian distribution (*μ*=0, *σ*=1) without any further statistical modifications. Consequently, the mean differences in miRNA expression among cases and controls were analyzed through a nonparametric test. This allowed us to, at first, identify BC-related miRNAs. This initial statistical analysis resulted in 40 miRNA candidates differentially expressed between BC cases and controls (Table 3). From these 40 candidate miRNAs, only miR-18a-5p, miR-372-3p, and miR-652-3p were highly expressed in controls rather than in BC cases, while the remaining 37 miRNAs were overexpressed in BC cases (Table 3).

**Figure 1:**
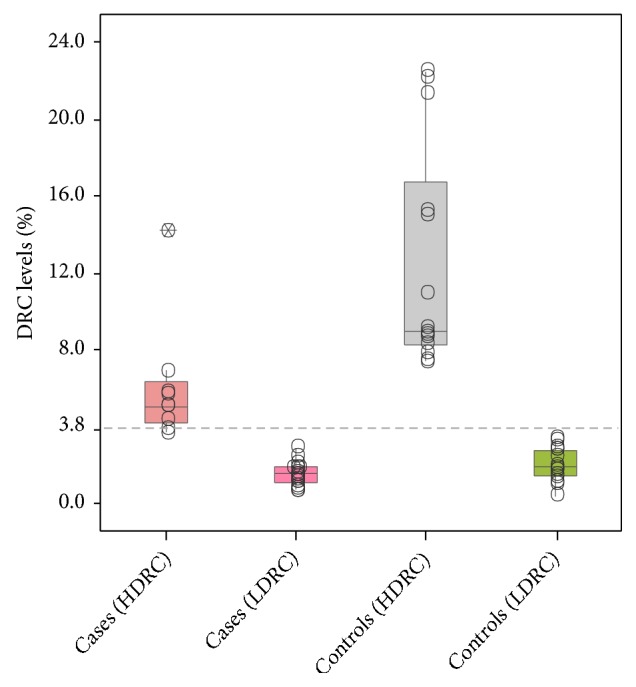
Distribution of DNA repair capacity levels among study participants including breast cancer cases and control. Groups were stratified into low (<3.8%) and high (≥3.8%) DRC based on a previously established cut-off (dotted line). Study groups were composed of BC cases with low (n=15) and high (n=14) DRC along with controls with low (n=18) and high (n=9) DRC levels. Box plots represent the data distribution of 26 breast cancer patients and 27 controls.

**Figure 2:**
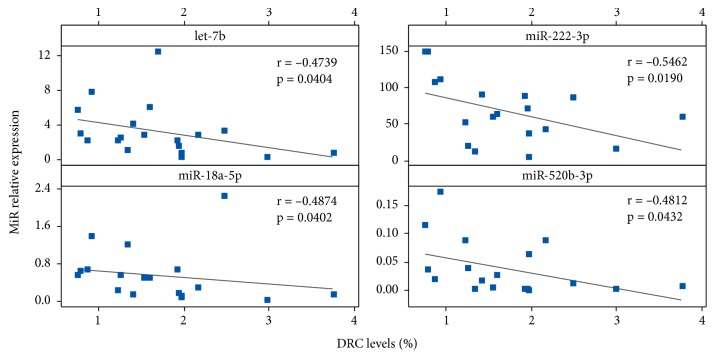
Correlation between selected microRNAs and DNA repair capacity levels. Linear regressions were performed to test for correlations between DRC levels and let-7b, miR-222-3p, miR-18a-5p, and miR-520-3p expression in BC patients (n=15). Blue squares represent BC patients with low DRC only. Correlations were tested using Spearman's rank correlation coefficient (p<0.05).

In order to identify miRNAs related to the overall DRC levels, BC cases and controls were stratified into low (<3.8%) and high (≥3.8%) DRC (Figure 1). Correlation analyses were performed focusing on low DRC BC cases only (Figure 2). Negative correlations were found between let-7b, miR-222-3p, miR-18a-5p, and miR-520-3p relative expression and DRC levels below the cut-off point of 3.8% (p<0.05, Spearman's correlation) (Figure 2).

### 3.3. DNA Repair Capacity-Related MiRNAs

**Figure 3:**
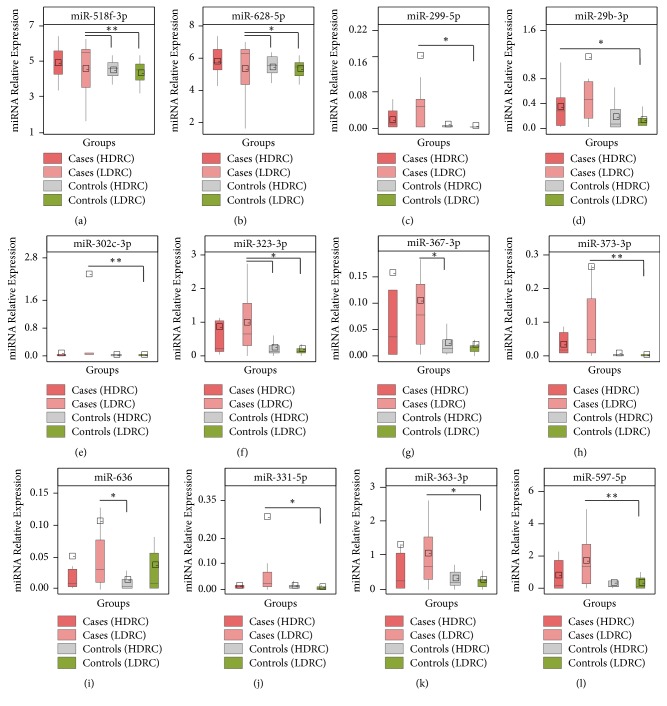
Comparison of relative microRNA expression between breast cancer cases and controls stratified by DRC. Groups were stratified into low (<3.8%) and high (≥3.8%) DRC based on a previously established cut-off. BC cases with low (n-=15) and high (n=14) DRC along with controls with low (n=18) and high (n=9) DRC levels were included in all panels. MicroRNAs were divided into 12 panels depending on their relative expression ranges. (a, b) miRNAs with extremely high relative expression were reported using a logarithmic scale. (c-l) miRNAs with a relative expression range 0-4. Each panel shows the miRNA relative expression distribution after normalization using the mammalian U6 endogenous control. Box plots represent the data distribution of 26 breast cancer cases and 27 controls. The point within the empty square represents the mean miRNA expression. DRC stratifications are represented by colors as can be seen in the legend (top). All miRNAs presented were differentially expressed among groups when mean comparisons were performed using KW test (p<0.05) followed by Dunn's multiple comparisons post hoc test (Table 3).

Differential expression of the 40 BC-related candidates was tested for relevance to DRC levels in BC cases and controls stratified by DRC levels using a Kruskal–Wallis (KW) test. To assess mean differences in miRNA expression among groups stratified by DRC as a dichotomous variable, a post hoc test was performed (Table 3). The following miRNAs were differentially expressed among the four study groups: miR-518f-3p, miR-628-5p, miR-299-5p, miR-29b-3p, miR-302c-3p, miR-323-3p, miR-367-3p, miR-373-3p, miR-636, miR-331-5p, miR-363-3p, and miR-597-5p (Figure 3). MicroRNAs with a high relative expression, miR-518f-3p and miR-628-5p, were plotted using a logarithmic scale (Figures 3(a) and 3(b)). For BC cases with low DRC levels, the mean expression of miR-628-5p was higher than the expression of miR-518f-3p (4.56% vs. 5.35%). The median of the low DRC BC cases shows a skewed distribution for both miRNAs (miR-518f-3p and miR-628-5p) which reveals the presence of biological outliers. In contrast, BC cases with high DRC had similar values for the median and the mean indicating a possible symmetric distribution (Figures 3(a) and 3(b)). The mean expression of these miRNAs in the control groups was similar independently of the DRC levels. In terms of the controls with low and high DRC, miR-299-5p, miR-302c-3p, miR-373-3p, and miR-331-5p showed a similar distribution in both groups (Figures 3(c), 3(e), 3(h), and 3(j)). In contrast, for miR-29b-3p, miR-323-3p, miR-367-3p, miR-636, and miR-597-5p, at least one of the control groups shows a slightly skewed distribution (Figures 3(d), 3(f), 3(g), 3(i), and 3(l)).

Relative miRNA expression was higher in BC cases than in controls for all miRNA candidates included in this analysis focused on DRC levels (Figures 3 and S2). Interestingly, some miRNAs were highly expressed in BC cases with high DRC such as miR-323-3p, miR-367-3p, and miR-363-3p (Figures 3(f), 3(g), and 3(k)). A different group of miRNAs had a higher expression in BC cases with low DRC levels, including miR-299-5p, miR-29b-3p, miR-302c-3p, miR-373-3p, miR-636, miR-331-5p, and miR-597-5p (Figures 3(c)–3(e), 3(h)–3(j), and 3(l)). When performing multiple comparison tests, significant differences in expression were found between BC cases and controls with low DRC for miR-299-5p (p<0.05), miR-302c-3p (p<0.01), miR-331-5p (p<0.05), miR-363-3p (p<0.05), miR-373-3p (p<0.01), and miR-597-5p (p<0.01) (Table 3 and Figure 3). Also, significant differences were observed between high DRC BC cases and low DRC controls for miR-29b-3p (p<0.05) and miR-518f-3p (p<0.01) (Table 3 and Figure 3). Only for miR-636, significant differences were observed between the BC cases and controls with high DRC (p<0.05).

Other differentially expressed candidates were miR-155-5p, miR-194-5p, miR-222-3p, miR-372-3p, miR-483-5p, and miR-342-3p; however, the post hoc test did not yield statistically significant results (p>0.05) (Figure S2). BC cases with low DRC had the highest relative expression of miR-155-5p, miR-194-5p, miR-296-3p, and miR-483-5p among all study groups, followed by high DRC BC cases (Figures S2(a)–S2(c) and S2(e)). In contrast, low DRC BC cases had the highest relative expression among groups for miR-372-3p and miR-342-3p, followed by high DRC BC cases (Figure S2(d) and S2(f)). As for the control groups, similar expression of these miRNAs was observed in high and low DRC groups (Figure S2(a)–S2(f)).

### 3.4. Multivariate Analyses

**Figure 4:**
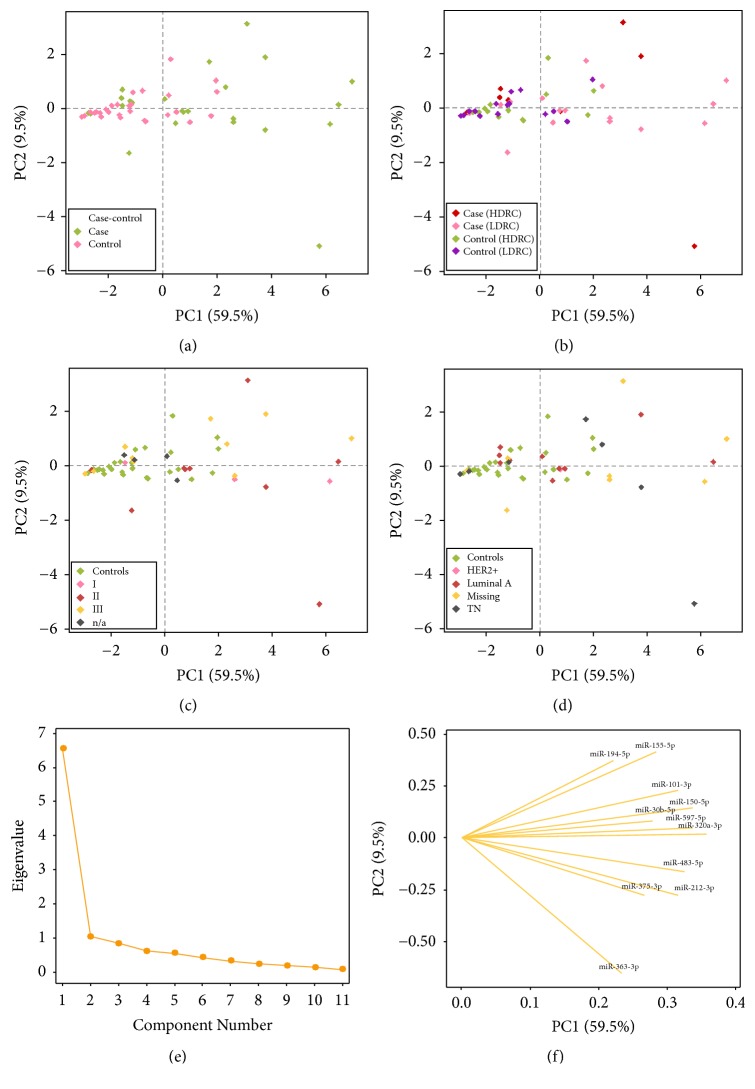
Population characteristics based on PCA matrix. The multivariate PCA matrix was used to localize the samples based on different stratifications: (a) case or control, (b) DRC (high and low), (c) tumor grade, and (d) BC subtypes. (e) PCA scree plot for the relative miRNAs expression of 26 BC cases and 27 controls. (f) Loading plot of miRNAs illustrating the PC1 and PC2 contribution to the variation among samples.

The principal component (PC) matrix was constructed using the miRNAs that were detected in all the samples, excluding miRNAs with missing values. The aim of this analysis was to identify the miRNAs responsible for the variation in expression among samples, as illustrated in Figure S1. A second aim was to identify any clustering using different variables such as case or control classification, DRC levels, tumor grade, and BC subtype. Among the miRNAs that qualified for this analysis were miR-101-3p, miR-150-5p, miR-155-5p, miR-194-5p, miR-212-3p, miR-30b-5p, miR-320a-3p, miR-363-3p, miR-375-3p, miR-483-5p, and miR-597-5p. The eigen analysis of the correlation matrix was used to generate the new PC coordinates with a cumulative variance of 0.69 using the two components. A scree plot was also used to choose the PC using the Kaiser criterion (Figure 4(e)) [30]. The PC2 variance contribution was very small (9.5%), and the PC1 weights were stronger for almost all these candidates (59.5%). The biplot graph using the PC1 and PC2 coordinates was generated and further used to localize the samples that were stratified by case or control classification, DRC levels, tumor grade, and BC subtype, which are presented in a color-coded graph panel (Figures 4(a)–4(f)). In order to discriminate among the miRNAs that were responsible for these clusters, a loading plot was generated using the PC axis (Figure 4(f)). Among the identified miRNAs with greater contribution to the PC1 were miR-101-3p, miR-150-5p, miR-320a-3p, miR-483-5p, miR-212-3p and miR-597-5p. The PC2 was below zero for miR-320a-3p and miR-597-5p. MiR-483-5p and miR-212-3p had a negative value on the PC2. The minor contributors for the PC1 were miR-155-5p, miR-194-5p, miR-30b-5p, miR-363-3p, and miR-375-3p. MiR-155-5p and miR-194-5p had the largest values on the PC2, followed by miR-101-3p and miR-150-5p. Thus, these miRNAs were also the ones with the highest degree of accumulative variance.

The PC analysis revealed that BC cases with high DRC were similar to the controls (independently of their DRC levels) and slightly different from the BC cases with low DRC on the PC1 axis. In Figure 4(b), an overlap can be seen between high DRC BC cases and controls showing that these samples are similar in terms of miRNA expression. The clinicopathological variables that were evaluated to possibly explain the data variation exposed in the mean comparison analyses and on the hierarchical heatmap were tumor site (ductal, lobular, or mixed), type of cancer (in situ or invasive), tumor grade, and molecular subtype. Only three samples were classified as a lobular malignancy and one sample had mixed components (combination of ductal and lobular) (Table 2). Only one tumor sample was classified as in situ BC, which accounted for 3.7% of the BC cases included in this pilot study. However, no clustering was detected in the PC analysis for tumor site and type of cancer when these samples were localized in the PC plot (data not shown). No clustering was observed on the PC analysis by tumor grade. Samples identified as grade I were spread along the PC1 axis. In addition, no clusters were identified when stratifiying samples by molecular BC subtype. However, the sample cohort did not have representation of the luminal B and HER2 subtypes.

## 4. Discussion

Although the role of miRNAs in BC has been extensively investigated and published, this pilot study is the first, to our knowledge, to establish a link between miRNA expression and overall DRC levels in BC cases. This study also represents one of the few assessing miRNA expression changes in Hispanic women with BC. Although a vast number of studies have been published regarding aberrant expression of miRNAs once the BC malignancy is developed, emerging evidence suggests that differences in miRNA expression profiles are partly influenced by ethnicity [19, 31, 32]. This pilot study provides new data on the miRNA expression profile of Hispanic women with BC and a basis for comparison of miRNAs profiles of women with BC from other populations. In addition, since the 27 women with BC studied were recently diagnosed and treatment naïve, treatment can be eliminated as a potential confounder.

### 4.1. Epidemiological and Clinicopathological Variables

The study cohort was composed of 56 Hispanic women from Puerto Rico where 51.8% were controls and 48.2% were BC cases. Epidemiological variables were categorized by DRC levels to assess differences among groups. Although no differences were observed for various epidemiological variables, a low DRC level was frequently observed in BC cases as we have previously published [19, 22, 33]. Overall, in our cohort, controls were younger than BC cases, as has been previously reported in many BC studies [1, 34]. Body mass index (BMI) has been reported to vary depending on age and ethnicity [19, 22, 35, 36]; however, in this study no association was found between BMI and having BC. Most of the participants reported having at least one pregnancy in the age range between 20 and 29 years. An equal proportion of the BC cases and controls (5.4%) reported being nulliparous. Early menarche (before age 12) is also a known BC risk factor [37]. Consistent with previous studies, we found an association between having the first menstrual period before 12 years old and having BC. Some surgical procedures have also been linked to decreasing BC risk (i.e., hysterectomy and oophorectomy); however, no association was found for any of these variables in our study group [38]. Lifestyle habits known to affect BC risk (i.e., smoking and alcohol consumption) were equally distributed in our cohort. No association with nonsporadic BC was found, based on the family history of BC and cancer in general.

Most of the BC cases were diagnosed with invasive ductal carcinoma including women with high and low DRC levels. Invasive ductal carcinoma is the most commonly diagnosed type of BC worldwide and this is reflected in our cohort [1, 39]. The tumor grade data was based on TNM staging system to classify the tumors among the 0, I, II, or III, or IV grade. Most of the BC cases had stage II and III tumors, independently of DRC levels. Ki-67 expression is used along with the molecular BC subtype classification for prognosis and to determine BC aggressiveness [40, 41]. Unfortunately, not enough data regarding Ki-67 expression was available; therefore, no further comparisons could be performed. No differences were found when stratifying cases by receptor status (ER, PR, and HER2) and DRC levels. The lack of association found between DRC levels and molecular subtypes was probably the result of a small sample size. In a recent study, involving 267 BC cases, we reported substantial variability in four molecular BC subtypes when analyzed in terms of DRC levels [16]. Consistent with that study, most of the women in the triple-negative BC group had low DRC levels. In general, the clinicopathological characteristics were equally distributed among groups.

### 4.2. Breast Cancer-Related MicroRNAs

MicroRNA expression was significantly different between cases and controls. A similar pattern has been reported in several studies [42–47]. The hierarchical matrix also revealed a characteristic pattern for every woman with and without BC based on relative miRNA expression. These unique patterns are also responsible for the variability observed in the mean comparison analyses and may be a reflection of biological variability among BC cases. Variations in plasma miRNA expression in BC have been reported for some of the 40 candidates identified as BC-related, including let-7b [48, 49], miR-155-5p [44, 50–52], miR-194-5p [53], miR-373-3p [54], and miR-375-3p [53]. Similar to our results, these miRNAs were overexpressed in the plasma from BC cases when compared to controls. Of these 40 candidates, only three miRNAs were underexpressed in BC cases: miR-18a-5p, miR-372-3p, and miR-652-3p. As for miR-18a-5p, a study by Jurkovicova et al. (2017) assessed the expression of several miRNAs including this miRNA in invasive and noninvasive BC cases and controls [55]. Although no significant differences among groups were reported, their results show that women with noninvasive BC had the highest expression of miR-18a-5p while women with invasive BC and controls had similar expression [55]. As for miR-372-3p, no studies have elucidated its expression levels in plasma from women with BC. However, the role of this miRNA in BC has been studied in breast tumor tissues, where its expression is lower in tumors than in adjacent normal tissue [56]. As for miR-652-3p, a recent study by Cuk et al. (2017) reported a higher expression of this miRNA in the plasma from women with BC when compared to women without BC [43].

### 4.3. MicroRNAs and DNA Repair in Breast Cancer

MicroRNAs regulate multiple genes involved in different DNA repair mechanisms [57]. Our pilot study provides a link between specific miRNAs and DRC levels (low and high), specifically through the NER pathway measured in lymphocytes. Very few studies have been aimed at elucidating the relationship between plasma miRNA expression and DRC in BC. Most of the studies aimed at elucidating this relationship have been performed in tumor tissues or using in vitro models. Initially, we identified four miRNAs that were negatively correlated with DRC within the range of low DRC levels: let-7b, miR-18a-5p, miR222-3p, and miR-520-3p. High let-7b expression has been associated with BC patients with high DRC; this is the first time that a negative correlation is detected in patients with low DRC [19]. In contrast with our results and as previously mentioned, miR-18a has been found to be upregulated in BC [55] and other cancer types [58]. Also, mechanistic studies show that miR-18a has an important role in downregulating ATM (Ataxia Telangiectasia Mutated), a DNA repair protein, in breast tumor tissues [59]. This can partially explain the negative correlation between miR-18a-5p and DRC levels. miR-222-3p has been widely studied in BC [60, 61] and tamoxifen resistance [62]. Moreover, this miRNA has been linked to DNA damage response by repressing RAD51 expression in ovarian cancer cells [63]. As for miR-520-3p, no reports on its expression changes in BC or any relationship with DRC were found. Among the 18 candidates found to be DRC-related, only miR-299-5p and miR-373 have been linked mechanistically to DNA repair. The study of Yan and coworkers shows that miR-299-5p expression is inversely correlated with RAD21 expression [64]. RAD21 is a protein involved in double-strand break repair [65]. A mechanistic study by Crosby and coworkers found that the forced expression of miR-373 induces a reduction in NER proteins, RAD23B and RAD52, in the breast cancer cell line MCF-7 [66].

### 4.4. Multivariate Analyses

Due to the variation observed in the heatmap matrix, a multivariate analysis was performed to study data variability using the following stratifications: case or control classification, DRC levels (high and low), tumor grade, and molecular subtype classification. The DRC levels and case-control stratifications were the best variables to describe the data variability, as described in the Results. Other studies have been performed using the PC algorithm to explain and reduce the biological variability. Wei et al. (2018) used this model to study 1046 miRNAs in tumors from esophageal cancer patients. Their results showed the entire variation of the data using 6 components [67]. Sredni et al. (2011) also used this method to study miRNAs extracted from whole blood from women stratified by age. This group illustrated a PC analysis that covered 40.8% of the data variation [68]. However, our PC model accounts for 69.0% of the data variability, with only two components. Although, two components are not an accurate representation of this data because they do not cover the entire variability of the data, it is the best approximation based on the eigenvalue graph. It is also important to highlight the fact that the miRNAs included in the PC matrix were significantly expressed between the cases and controls and, thus, are BC-related in our cohort. The PC analysis demonstrated that the DRC levels can be related to the data variability. Our results indicate that miR-101-3p, miR-150-5p, miR-320a-3p, miR-483-5p, miR-212-3p, and miR-597-5p are responsible for this sample variability.

## 5. Conclusions

In conclusion, we identified 40 BC-related miRNAs that may have an important role in the epigenetics of Hispanic BC patients. This pilot study provides evidence of four miRNAs that are negatively regulated in BC cases with low DRC levels. Finally, the PC analysis suggested that the clinicopathological characteristics may not have a direct effect on specific miRNA expression. Additional studies are needed in order to have a complete framework regarding the overall DRC levels, miRNA expression profiles, and the tumor characteristics. When our results are validated with a larger sample size, this knowledge will become a pivotal force to study prognosis, recurrence, and treatment outcomes based on the overall DRC levels.

## Figures and Tables

**Table 1:** Description of the study group including DNA repair capacity levels and selected breast cancer risk factors in cases and controls.

Epidemiological characteristics	Controls	BC cases	p-value
	n=29 (%)	n=27 (%)	
*DRC*			
Low (<3.8%)	15 (26.8)	18 (32.1)	0.2561
High (≥3.8%)	14 (25.0)	9 (16.1)	
*Age*			
21-40	7 (12.5)	0 (0.0)	0.0239
41-60	11 (19.6)	14 (25.0)	
61+	11 (19.6)	13 (23.2)	
*BMI*			
<25 kg/m^2^	14 (25.0)	9 (16.1)	0.2772
≥25 kg/m^2^	14 (25.0)	18 (32.1)	
Missing	1 (1.8)	0 (0.0)	
*Ever been pregnant*			
Yes	26 (46.4)	24 (42.9)	0.9262
No	3 (5.4)	3 (5.4)	
*Age at first birth*			
≤19	6 (10.7)	4 (7.1)	0.6075
20-29	14 (25.0)	16 (28.6)	
≥30	6 (10.7)	2 (3.6)	
Missing	0 (0.0)	2 (3.6)	
*Parity*			
Nulliparous	3 (5.4)	3 (5.4)	0.2244
1-2 children	15 (26.8)	8 (14.3)	
≥3 children	11 (19.6)	16 (28.6)	
*Ever breastfeed*			
Yes	15 (26.8)	11 (19.6)	0.4102
No	14 (25.0)	16 (28.6)	
*Length of breastfeeding*			
Never	14 (25.0)	11 (19.6)	0.0585
1-5 months	13 (23.2)	11 (19.6)	
≥6 months	2 (3.6)	0 (0.0)	
Missing	0 (0.0)	5 (8.9)	
*Oral contraceptive use*			
Yes	13 (23.2)	8 (14.3)	0.2405
No	16 (28.6)	19 (33.9)	
*Age started oral contraceptive*			
<20	2 (3.6)	1 (1.8)	1
≥21	11 (19.6)	7 (12.5)	
*Regular menstrual periods*			
Yes	15 (26.8)	19 (33.9)	0.1534
No	14 (25.0)	8 (14.3)	
*Age Menarche*			
≤12	0 (0.0)	11 (19.6)	0.0001
≥13	26 (46.4)	14 (25.0)	
Missing	3 (5.4)	2 (3.6)	
*History of endometriosis*			
Yes	3 (5.4)	1 (1.8)	0.3349
No	26 (46.4)	26 (46.4)	
*Hysterectomy*			
Yes	9 (16.1)	6 (10.7)	0.4568
No	20 (35.7)	21 (37.5)	
*Age of hysterectomy*			
≤40	6 (10.7)	1 (1.8)	0.1264
41-49	1 (1.8)	3 (5.4)	
≥50	2 (3.6)	2 (3.6)	
*Oophorectomy*			
Yes	8 (14.3)	6 (10.7)	0.5589
No	20 (35.7)	21 (37.5)	
Missing	1 (1.8)	0 (0.0)	
*Age of oophorectomy*			
≤40	4 (7.1)	2 (3.6)	0.1264
41-49	2 (3.6)	2 (3.6)	
≥50	2 (3.6)	2 (3.6)	
*Menopause (actually)*			
Yes	4 (7.1)	3 (5.4)	0.7664
No	23 (41.1)	22 (39.3)	
Missing	2 (3.6)	2 (3.6)	
*Hormone replacement therapy*			
Yes	14 (25.0)	6 (10.7)	0.0420
No	15 (26.8)	21 (37.5)	
*Smoking*			
Yes	1 (1.8)	3 (5.4)	0.2659
No	28 (50.0)	24 (39.3)	
*Alcohol consumption*			
Yes	5 (8.9)	5 (8.9)	0.9008
No	24 (42.9)	22 (39.3)	
*Family history of cancer (not BC)*			
Yes	13 (23.2)	13 (23.3)	0.8034
No	16 (28.6)	14 (25.0)	
*BC history in any family member*			
Yes	16 (28.6)	12 (21.4)	0.4224
No	13 (23.2)	15 (26.8)	

Pearson's chi-square test was performed to assess significance among groups.

DRC: DNA repair capacity, BMI: body mass index, BC: breast cancer.

**Table 2:** Clinicopathological characteristics of BC cases with low and high levels of DNA repair capacity.

Clinicopathological Characteristics	Low DRC (<3.8%)	High DRC (≥3.8%)	p-value
	n=18 (%)	n=9 (%)	
*Estrogen receptor status*			
Positive	9 (33.3)	3 (11.1)	0.5698
Negative	6 (22.2)	3 (11.1)	
Missing	3 (11.1)	3 (11.1)	
*Progesterone receptor status*			
Positive	8 (29.6)	3 (11.1)	0.6121
Negative	7 (25.9)	3 (11.1)	
Missing	3 (11.1)	3 (11.1)	
*HER2 status*			
Positive	0 (0.0)	1 (3.7)	0.7160
Negative	12 (44.4)	5 (18.5)	
Missing	6 (22.2)	3 (11.1)	
*Ki-67*			
Low (≤10%)	1 (3.7)	1 (3.7)	0.9297
Borderline (11-20%)	0 (0.0)	0 (0.0)	
High (≥21%)	3 (11.1)	1 (3.7)	
Missing	15 (55.6)	6 (22.2)	
*Grade*			
I	4 (14.8)	0 (0.0)	0.4194
II	6 (22.2)	4 (14.8)	
III	5 (18.5)	4 (14.8)	
Missing	3 (11.1)	1 (3.7)	
*Molecular Subtypes*			
Luminal A	7 (25.9)	3 (11.1)	0.6923
Luminal B	0 (0.0)	0 (0.0)	
HER2+	0 (0.0)	1 (3.7)	
Triple-negative	5 (18.5)	2 (7.4)	
Missing	6 (22.2)	3 (11.1)	
*Site*			
Ductal	16 (59.3)	7 (25.9)	0.3522
Lobular	2 (7.4)	1 (3.7)	
Ductal + Lobular	0 (0.0)	1 (3.7)	
*Type*			
*In situ*	1 (3.7)	0 (0.0)	0.4712
Invasive	17 (63.0)	9 (33.3)	

Pearson's chi-square test was performed to assess significance among groups.

DRC: DNA repair capacity, BMI: body mass index, BC: breast cancer.

**Table 3:** Breast cancer-related miRNAs and comparisons with women with low and high DRC levels.

miRNA ID	Case-Control	Regulation	DRC	Multiple Comparisons*∗*	*p-value* ^*3*^
	Groups^1^		Groups^2^		
let-7b	0.0153	↑	0.1062	-	*-*
let-7c	0.0157	↑	0.0581	-	*-*
let-7e	0.0477	↑	0.2243	-	*-*
miR-101-3p	0.0389	↑	0.1517	-	*-*
miR-150-5p	0.0258	↑	0.1615	-	*-*
miR-155-5p	0.0043	↑	0.0329	NS	*p>0.05*
miR-18a-5p	0.0205	↓	0.1344	-	*-*
miR-194-5p	0.0083	↑	0.0465	NS	*p>0.05*
miR-204-5p	0.0337	↑	0.1175	-	*-*
miR-212-3p	0.0227	↑	0.0857	-	*-*
miR-222-3p	0.0330	↑	0.1847	-	*-*
miR-25-3p	0.0455	↑	0.1010	-	*-*
miR-296-3p	0.0440	↑	0.0322	NS	*p>0.05*
miR-299-5p	0.0143	↑	0.0362	BC LDRC vs. Controls LDRC	*∗p<0.05*
miR-29b-3p	0.0021	↑	0.0168	BC HDRC vs. Controls LDRC	*∗p<0.05*
miR-302a-3p	0.0163	↑	0.0616	-	*-*
miR-302c-3p	0.0022	↑	0.0040	BC LDRC vs. Controls LDRC	*∗∗p<0.01*
miR-30b-5p	0.0258	↑	0.1107	-	*-*
miR-320a-3p	0.0492	↑	0.2296	-	*-*
miR-323-3p	0.0002	↑	0.0014	BC cases LDRC vs. Controls (LDRC & HDRC)	*∗p<0.05*
miR-331-5p	0.0124	↑	0.0145	BC LDRC vs. Controls LDRC	*∗p<0.05*
miR-342-3p	0.0065	↑	0.0297	NS	*p>0.05*
miR-363-3p	0.0227	↑	0.0394	BC LDRC vs. Controls LDRC	*∗p<0.05*
miR-367-3p	0.0046	↑	0.0235	BC LDRC vs. Controls HDRC	*∗p<0.05*
miR-372-3p	0.0024	↓	0.0254	NS	*p>0.05*
miR-373-3p	0.0006	↑	0.0077	BC LDRC vs. Controls LDRC	*∗∗p<0.01*
miR-375-3p	0.0175	↑	0.1060	-	*-*
miR-383-5p	0.0135	↑	0.0849	-	*-*
miR-425-5p	0.0165	↑	0.0540	-	*-*
miR-483-5p	0.0121	↑	0.0386	NS	*p>0.05*
miR-486 -5p	0.0234	↑	0.1370	-	*-*
miR-509-5p	0.0226	↑	0.1330	-	*-*
miR-518f-3p	0.0002	↑	0.0011	BC cases LDRC vs. Controls (LDRC&HDRC)	*∗∗p<0.01*
miR-520b-3p	0.0084	↑	0.0615	-	*-*
miR-525-5p	0.0055	↑	0.0537	-	*-*
miR-597-5p	0.0029	↑	0.0073	BC LDRC vs. Controls LDRC	*∗∗p<0.01*
miR-628-5p	0.0003	↑	0.0026	BC cases LDRC vs. Controls (LDRC&HDRC)	*∗p<0.05*
miR-636	0.0205	↑	0.0266	BC cases LDRC vs. Controls HDRC	*∗p<0.05*
miR-652-3p	0.0423	↓	0.2382	-	*-*
miR-708-5p	0.0187	↑	0.1298	-	*-*

*p-value* ^*1*^ *:* obtained from Mann–Whitney test (BC case-control comparisons); *p-value*^*2*^: obtained from Kruskal–Wallis test (DRC stratifications); *p-value*^*3*^: obtained from Dunn's multiple comparisons post hoc test. NS: nonsignificant, BC: breast cancer, LDRC: low DNA repair capacity, HDRC: high DNA repair capacity. Arrows represent up- or downregulation in BC cases when compared to controls.

**∗** means groups significantly different from post hoc analysis.

## Data Availability

The data used to support the findings of this study are available from the corresponding author upon request.

## Supplementary Materials

Supplementary Materials Figure S1: hierarchical MicroRNA clustering. After case-control comparisons using a Mann–Whitney U test, 40 candidate microRNAs were obtained. Left side (a) shows the expression profile for BC cases (X1-X.26). Right side (b) shows the miRNA expression for the controls (X2-X.29). Table S1: target sequences for the microRNAs included in this study. Figure S2: comparison of relative microRNA expression between breast cancer cases and controls stratified by DRC. BC cases with low (n-=15) and high (n=14) DRC along with controls with low (n=18) and high (n=9) DRC levels were included in all panels. Each panel shows the miRNA relative expression distribution after normalization using the mammalian U6 endogenous control. Box plots represent the data distribution of 26 breast cancer patients and 27 controls. The point within the empty square represents the mean miRNA expression. DRC stratifications are represented by colors. All miRNAs presented were differentially expressed among groups when mean comparisons were performed using KW test (p<0.05) only (Table 3).
