# Supplementary material for: MicroRNA Expression Changes in Women with Breast Cancer Stratified by DNA Repair Capacity Levels
Source: J Oncol. 2019 May 5;2019:7820275. doi: 10.1155/2019/7820275 (PMC6525916; doi:10.1155/2019/7820275)
Supplement: Supplementary Materials — Figure S1: hierarchical MicroRNA clustering. After case-control comparisons using a Mann–Whitney U test, 40 candidate microRNAs were obtained. Left side (a) shows the expression profile for BC cases (X1-X.26). Right side (b) shows the miRNA expression for the controls (X2-X.29). Table S1: target sequences for the microRNAs included in this study. Figure S2: comparison of relative microRNA expression between breast cancer cases and controls stratified by DRC. BC cases with low (n-=15) and high (n=14) DRC along with controls with low (n=18) and high (n=9) DRC levels were included in all panels. Each panel shows the miRNA relative expression distribution after normalization using the mammalian U6 endogenous control. Box plots represent the data distribution of 26 breast cancer patients and 27 controls. The point within the empty square represents the mean miRNA expression. DRC stratifications are represented by colors. All miRNAs presented were differentially expressed among groups when mean comparisons were performed using KW test (p<0.05) only (Table 3). [file 7820275.f1.pdf]

Supplementary Materials

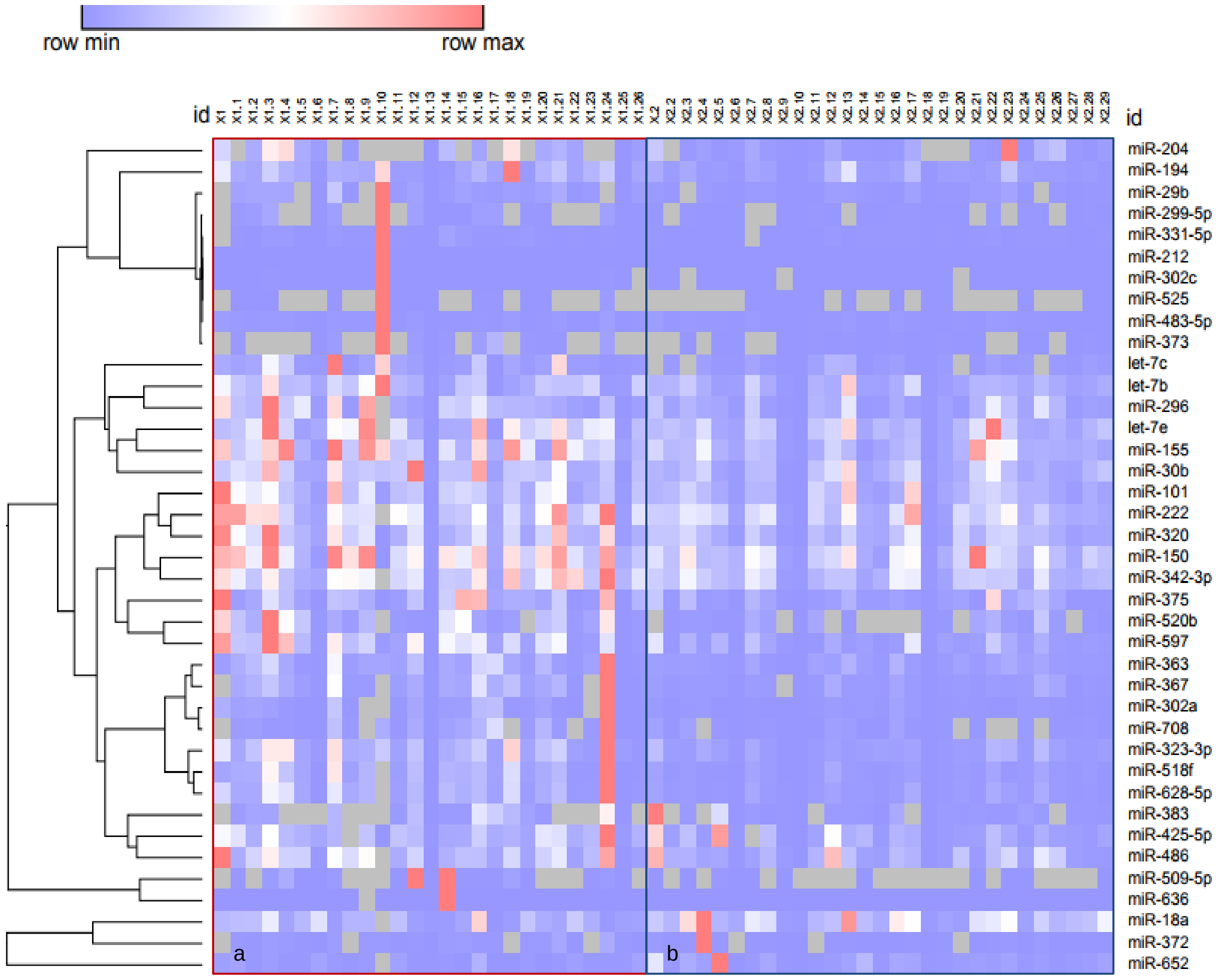

Figure S1: Hierarchical MicroRNA Clustering. After case-control comparisons using a Mann-Whitney U test, 40 candidate microRNAs were obtained. Left side (a) shows the expression profile for BC cases (X1-X.26). Right side (b) shows the miRNA expression for the controls (X2-X.29).

Table S1. Target sequences for the microRNAs included in this study.

miRNA ID	Target Sequence
let-7b	UGAGGUAGUAGGUUGUGUGGUU
let-7c	UGAGGUAGUAGGUUGUAUGGUU
let-7e	UGAGGUAGGAGGUUGUAUAGUU
miR-101-3p	UACAGUACUGUGAUAACUGAA
miR-150-5p	UCUCCCAACCCUUGUACCAGUG
miR-155-5p	UUAAUGCUAAUCGUGAUAGGGGU
miR-18a-5p	UAAGGUGCAUCUAGUGCAGAUAG
miR-194-5p	UGUAACAGCAACUCCAUGUGGA
miR-204-5p	UUCCCUUUGUCAUCCUAUGCCU
miR-212-3p	UAACAGUCUCCAGUCACGGCC
miR-222-3p	AGCUACAUCUGGCUACUGGGU
miR-25-3p	CAUUGCACUUGUCUCGGUCUGA
miR-296-3p	GAGGGUUGGGUGGAGGCUCUCC
miR-299-5p	UGGUUUACCGUCCCACAUACAU
miR-29b-3p	UAGCACCAUUUGAAAUCAGUGUU
miR-302a-3p	UAAGUGCUUCCAUGUUUUGGUGA
miR-302c-3p	UAAGUGCUUCCAUGUUUCAGUGG
miR-30b-5p	UGUAAACAUCCUACACUCAGCU
miR-320a-3p	AAAAGCUGGGUUGAGAGGGCGA
miR-323-3p	CACAUUACACGGUCGACCUCU
miR-331-5p	CUAGGUAUGGUCCCAGGGAUCC
miR-342-3p	UCUCACACAGAAAUCGCACCCGU
miR-363-3p	AAUUGCACGGUAUCCAUCUGUA
miR-367-3p	AAUUGCACUUUAGCAAUGGUGA
miR-372-3p	AAAGUGCUGCGACAUUUGAGCGU
miR-373-3p	GAAGUGCUUCGAUUUUGGGGUGU
miR-375-3p	UUUGUUCGUUCGGCUCGCGUGA
miR-383-5p	AGAUCAGAAGGUGAUUGUGGCU
miR-425-5p	AAUGACACGAUCACUCCCGUUGA
miR-483-5p	AAGACGGGAGGAAAGAAGGGAG
miR-486 -5p	UCCUGUACUGAGCUGCCCCGAG
miR-509-5p	UACUGCAGACAGUGGCAAUCA
miR-518f-3p	GAAAGCGCUUCUCUUUAGAGG
miR-520b-3p	AAAGUGCUUCCUUUUAGAGGG
miR-525-5p	CUCCAGAGGGAUGCACUUUCU
miR-597-5p	UGUGUCACUCGAUGACCACUGU
miR-628-5p	AUGCUGACAUAUUUACUAGAGG
miR-636	UGUGCUUGCUCGUCCCGCCCGCA
miR-652-3p	AAUGGCGCCACUAGGGUUGUG
miR-708-5p	AAGGAGCUUACAAUCUAGCUGGG

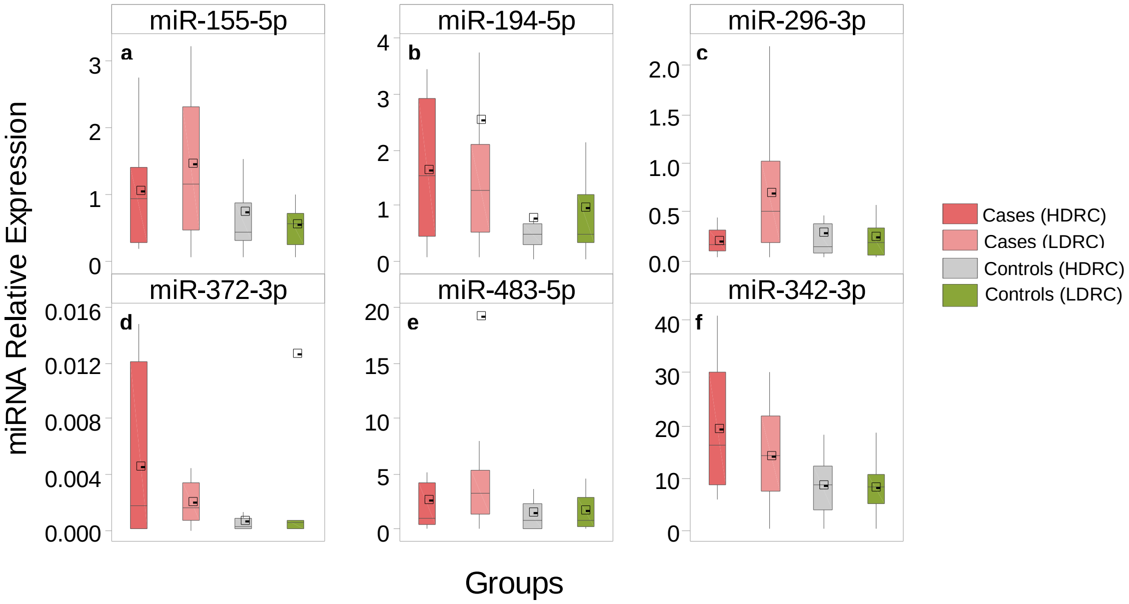

Figure S2: Comparison of relative microRNA expression between breast cancer cases and controls stratified by DRC. BC cases with low (n-=15) and high (n=14) DRC along with controls with low (n=18) and high (n=9) DRC levels were included in all panels. Each panel shows the miRNA relative expression distribution after normalization using the mammalian U6 endogenous control. Box plots represent the data distribution of 26 breast cancer patients and 27 controls. The point within the empty square represents the mean miRNA expression. DRC stratifications are represented by colors. All miRNAs presented were differentially expressed among groups when mean comparisons were performed using KW test ($p<0.05$) only (Table 3).
